# Management of Apatinib-Related Adverse Events in Patients With Advanced Osteosarcoma From Four Prospective Trials: Chinese Sarcoma Study Group Experience

**DOI:** 10.3389/fonc.2021.696865

**Published:** 2021-07-22

**Authors:** Lu Xie, Jie Xu, Wei Guo, Zhen Wang, Yang Yao, Jianmin Li, Jianhua Lin, Jianru Xiao, Xiuchun Yu, Weibin Zhang, Zhendong Cai, Yingqi Hua, Jing Chen, Zengwu Shao, Di Wu, Sujia Wu, Zhongqi Tu, Xiaojing Zhang

**Affiliations:** ^1^ Musculoskeletal Tumor Center, Peking University People’s Hospital, Beijing, China; ^2^ Orthopedic Oncology, Xijing Hospital Air Force Medical University of PLA (The Fourth Military Medical University), Xi’an, China; ^3^ Medical Oncology, Shanghai Sixth People’s Hospital, Shanghai, China; ^4^ Orthopedic Oncology, Qilu Hospital of Shandong University, Jinan, China; ^5^ Musculoskeletal Tumor Center, The First Affiliated Hospital of Fujian Medical University, Fuzhou, China; ^6^ Orthopedic Oncology, Shanghai Changzheng Hospital, Shanghai, China; ^7^ Orthopedic Oncology, Jinan Military General Hospital, Jinan, China; ^8^ Orthopedic Oncology, Ruijin Hospital Affiliated to Shanghai Jiaotong University, Shanghai, China; ^9^ Orthopedic Oncology, Shanghai General Hospital, Shanghai, China; ^10^ Orthopedic Oncology and Medical Oncology, Wuhan Union Hospital Affiliated to Tongji Medical College of Huazhong University of Science and Technology, Wuhan, China; ^11^ Medical Oncology, The First Affiliated Hospital of Jilin University, Changchun, China; ^12^ Orthopedic Oncology, General Hospital of Eastern Theater Command, Nanjing, China; ^13^ Orthopedic Oncology, Huaxi Hospital West China School of Medicine/West China Hospital of Sichuan University (WCSM/WCH), Chengdu, China; ^14^ Musculoskeletal Tumor Center, Liaoning Cancer Hospital & Institute, Shenyang, China

**Keywords:** apatinib, toxicity, prophylaxis, treatment, osteosarcoma

## Abstract

Four prospective trials have reported apatinib-related efficacy in osteosarcoma, with a high response rate of 43.2%. Currently, Adverse Events (AEs) have increasingly gained attention, as treatment with multiple tyrosine kinase inhibitors (TKIs) is potentially lifelong. For this reason, a consensus meeting of the Chinese Sarcoma Study Group (CSSG), which is a multidisciplinary panel composed of pediatric, medical and surgical oncologists specializing in sarcoma, nurse specialists, oncological senior pharmacists and gastroenterologists, was held to develop comprehensive guidelines on AEs emerging due to apatinib treatment to better assist in the prevention, management, and understanding of AE development. We summarized all AEs that arose in ≥10% of the participants as well as rare AEs that required extra caution to prevent that were observed in these four published prospective trials and arranged these AEs into 14 disorder systems according to CTCAE 5.0. In this review, we discuss strategies for the management of AEs in patients with advanced osteosarcoma, with the aim of maximizing treatment benefits and minimizing the need for apatinib treatment discontinuation. We also focus on providing recommendations for the prophylaxis and treatment of advanced osteosarcoma using apatinib to achieve optimal outcomes.

## Introduction

Osteosarcoma has a five-year overall survival of 71% (95% CI: 68%−73%) based on the European and American Osteosarcoma Study (EURAMOS-1) trial (1–3). However, treatment options for chemotherapy refractory cases remain limited, and prognosis is dismal. Tyrosine kinase inhibitors (TKIs) targeting angiogenesis, such as vascular endothelial growth factor receptors (VEGFRs), have been verified to be helpful in prolonging the progression-free survival (PFS) of advanced osteosarcoma patients who progressed on chemotherapy (4, 5). Apatinib, a specific TKI that mainly targets VEGFR-2, has been investigated in patients with advanced osteosarcoma (6). The combination of apatinib with camrelizumab, a high-affinity, humanized,IgG4-κ PD-1 monoclonal antibody (7), has resulted in the prolongation of PFS and two durable responses (8).

However, more than half of our enrolled patients had a dose reduction or temporal interruption due to toxicity (6, 7, 9, 10). In real-world practice, at least a quarter of patients change their TKIs at least once during their lifetime because of drug intolerance (11–13). Although the tumor burden can be significantly reduced by apatinib, the quality of life (QoL) of patients was not improved, which seems to place apatinib in the same situations as sorafenib, sunitinib, regorafenib, and pazopanib (14–17). The need for the continuous daily use of apatinib until secondary resistance is hampered by the associated long-term adverse events (AEs) and the resulting decrease in QoL. The attention given by the Chinese Sarcoma Study Group (CSSG) to AEs has grown in the past few years, but our understanding remains poor. Furthermore, we noticed that publications on the prevention and management of apatinib-related AEs were limited (18, 19). Thus, the CSSG working party asked authors LX, JX and WG to convene a group of physicians or medical workers who had previously published and/or taken part in apatinib-related trials or frequently used this drug in the clinic and reported their findings at local meetings. These people were asked to retrospectively investigate all valuable methods in relieving the toxicities of apatinib and to provide medical advice on when apatinib is the appropriate treatment regimen and when it should be avoided. This publication provides a consensus on the use of apatinib based on discussions by email, as well as discussions at a meeting held in Dalian, China on August 30, 2019. Here, we review the tolerability profiles of apatinib during the treatment of advanced osteosarcoma, mainly focusing on AE management strategies.

## AEs During Apatinib-Related Therapy

The Peking University People’s Hospital Sarcoma Group (PKUPH-sarcoma) study is a single-arm, nonblind, phase-two study that evaluated the efficacy and safety of apatinib in patients with ≥16 years of advanced osteosarcoma progressing on chemotherapy and is registered at ClinicalTrials.gov (NCT02711007) (6). Another single-arm phase II trial (NCT03121846) was designed to assess the biological activity of apatinib in relation to its efficacy and safety for bone sarcomas and soft tissue sarcomas and was conducted at Tianjin Medical University Cancer Institute & Hospital (9). The third study came from the Affiliated Cancer Hospital of Zheng Zhou University, Henan Cancer Hospital and was an open-label, nonrandomized, single-center study of 45 patients with bone and soft tissue sarcomas conducted from May 2017 to July 2018 (NCT03163381) (10). Apatinib plus camrelizumab (SHR-1210) for unresectable high-grade osteosarcoma (APFAO) is the fourth independent study investigating the administration of apatinib mesylate and camrelizumab for treating patients with inoperable locally advanced or metastatic osteosarcoma who progressed after chemotherapy (NCT03359018) (7). In general, drug-related AEs were limited to grade one or two. The frequency of apatinib administration (the total dose over time) was 40.5%-60% of the planned dose (6, 7, 9, 10). The most common grade 3 or 4 AEs are listed in **Table 1**.

**Table 1:** Adverse events that arose in the two apatinib-related trials (NCT02711007 and NCT03359018).

	Apatinib for Advanced Osteosarcoma (NCT02711007) (*N* = 37)		Apatinib and Camrelizumab for Advanced Osteosarcoma (NCT03359018) (*N* = 43)		Apatinib for Stage IV Sarcomas(NCT03121846)(N=64)		Apatinib for Advanced Bone and Soft Tissue Sarcomas(NCT03163381)(N=45)	
	All, *n* (%)	Grade 3/4, *n* (%)	All, *n* (%)	Grade 3/4, *n* (%)	All, *n* (%)	Grade 3/4, *n* (%)	All, *n* (%)	Grade 3/4, *n* (%)
**Cardiac disorders**								
Palpitations	3 (8.11)	1 (2.70)	5 (11.63)	0 (0.00)	2 (3.13)	0 (0.00)	NR^b^	NR
**Endocrine disorders**								
Hypoparathyroidism	8 (21.62)	0 (0.00)	9 (20.93)	1 (2.33)	NR	NR	NR	NR
**Gastrointestinal disorders**								
Abdominal pain	4 (10.81)	2 (5.41)	8 (18.60)	2 (4.65)	NR	NR	NR	NR
Anal mucositis, ulcer and hemorrhage	3 (8.10)	1 (2.70)	1 (2.33)	1 (2.33)	NR	NR	NR	NR
Diarrhea	7 (18.92)	3 (8.10)	21 (48.84)	2 (4.65)	7 (10.94)	0 (0.00)	16 (35.56)	2 (12.50)
Oral mucositis	4 (10.81)	0 (0.00)	11 (25.58)	2 (4.65)	5 (7.81)	0 (0.00)	NR	NR
Nausea and vomiting	0 (0.00)	0 (0.00)	10 (23.25)	1 (2.33)				
Toothache	0 (0.00)	0 (0.00)	6 (13.95)	2 (4.65)	3 (4.69)	0 (0.00)	NR	NR
**General disorders and administration site conditions**								
Fatigue	12 (32.43)	1 (2.7)	4 (9.30)	1 (2.33)	8 (12.50)	1 (1.56)	16 (35.56)	1 (6.25)
**Hepatobiliary disorders**								
Cholecystitis	1 (2.7)	1 (2.7)	1 (2.33)	0 (0.00)	NR	NR	NR	NR
**Injury, poisoning and procedural complications**								
Wound complications	4 (10.81)	4 (10.81)	11 (25.58)	6 (13.95)	NR	NR	NR	NR
**Investigations**								
Alanine/aspartate aminotransferase increases	1 (2.70)	0 (0.00)	18 (41.86)	2 (4.65)	4 (6.25)	0 (0.00)	NR	NR
Blood bilirubin increases	7 (18.92)	0 (0.00)	22 (51.16)	4 (9.30)	3 (4.69)	0 (0.00)	NR	NR
Electrocardiogram Prolonged QTc interval	0 (0.00)	0 (0.00)	3 (6.98)	0 (0.00)	NR	NR	NR	NR
Platelet count decreases	1 (2.70)	0 (0.00)	30 (69.77)	2 (4.65)	NR	NR	NR	1 (6.25)
Thyroid-stimulating hormone increases	8 (21.62)	3 (8.11)	35 (81.4)	1 (2.33)	NR	NR	NR	NR
Weight loss	12 (32.43)	1 (2.70)	15 (34.88)	3 (6.98)	NR	NR	NR	NR
White blood cell decreases	0 (0.00)	0 (0.00)	16 (37.21)	2 (4.65)	NR	NR	NR	NR
**Metabolism and nutrition disorders**								
Anorexia	13 (35.14)	2 (5.41)	20 (46.51)	3 (6.98)	14 (21.88)	0 (0.00)	NR	NR
Hyperlipidemia	10 (27.03)	1 (2.70)	32 (74.42)	3 (6.98)	NR	NR	NR	NR
Hypokalemia	3 (8.11)	1 (2.7)	17 (39.53)	0 (0.00)	NR	NR	NR	NR
**Musculoskeletal and connective tissue disorders**								
Pain in extremity	5 (13.51)	0 (0.00)	20 (46.51)	2 (4.65)	7 (10.94)	1 (1.56)	NR	NR
**Psychiatric disorders**								
Insomnia	2 (5.41)	0 (0.00)	2 (4.65)	0 (0.00)	NR	NR	NR	NR
**Renal and urinary disorders**								
Proteinuria	4 (10.81)	3 (8.11)	11 (25.58)	1 (2.33)	19 (29.69)	2 (3.13)	14 (31.11)	2 (14.29)
**Respiratory, thoracic and mediastinal disorders**								
Hoarseness and oropharyngeal pain	0 (0.00)	0 (0.00)	3 (6.98)	0 (0.00)	4 (6.25)	0 (0.00)	NR	NR
Pneumothorax	12 (32.43)	6 (16.22)	9 (20.93)	3 (6.98)	2 (3.13)	0 (0.00)	NR	NR
**Skin and subcutaneous tissue disorders**								
Hair color changes	1 (2.70)	0 (0.00)	5 (11.63)	0 (0.00)	5 (7.81)	0 (0.00)	NR	NR
Hand–foot skin reaction^a^	14 (37.84)	5 (13.51)	21 (48.84)	2 (4.65)	22 (34.38)	3 (4.69)	15 (33.33)	1 (6.67)
**Vascular disorders**								
Hypertension	7 (18.92)	0 (0.00)	10 (23.26)	2 (4.65)	24 (37.50)	5 (7.81)	17 (37.8)	2 (12.50)

a Hand–foot skin reactions including palmar-plantar erythrodysesthesia syndrome and rash maculo-papular.

b NR, not reported.

Tumor growth in relation to angiogenesis that is effectively targeted may be the cause of mechanism-induced toxicity but is also linked with efficacy (20, 21). Some TKI-associated AEs were reported to be correlated with better patient prognosis. In our single-agent apatinib trial for osteosarcoma, loss of appetite was also significantly related to longer PFS based on a Cox regression model (HR, 0.35; *p* = 0.01) (6). For the clinical benefit rate (CBR, CR+PR+SD for more than 6 months according to RECIST 1.1), patients who had pneumothorax and hypothyroidism showed prolonged PFS compared with those who did not (*p* = 0.07 and 0.00, respectively) (6). These AEs that arise early in the treatment course, which are reversible and alleviated by supportive care, would predict better outcomes. However, if they deteriorated to persistent dose reductions, drug efficacy might be compromised afterwards.

## Manifestation and Management of AEs

Over the last decade, a wealth of strategies have been developed for the management of TKI-associated AEs in other solid tumors. Most of these strategies can be used in the management of AEs associated with apatinib (18, 19). Before using this drug, it is important to determine whether the study participants have a medical history of hypertension, cerebrovascular disease, thrombosis and hemorrhage, heart disease, or even history of AEs using other antiangiogenic TKIs because this would help prevent possible severe AEs later. In addition, monitoring some laboratory parameters at baseline and during treatment (e.g., routine blood tests, hepatorenal functions, urine protein, and thyroid function) and other systems (e.g., cardiovascular, cutaneous, mucosal, and neurological systems) may also prevent the development of severe AEs.

**Table 2** shows the recommended dose reductions of apatinib during treatment. Comprehensive recognition in the early stage of treatment is crucial for optimal prophylaxis in the case of inevitably compromised treatment continuity. Most of the study protocols have used the National Cancer Institute Common Terminology Criteria for Adverse Events (NCI-CTCAE) for classifying the severity of AEs, and there are similarities in management strategies across different tumors. It is worth noting that the NCI-CTCAE criteria are not always comprehensive enough to describe the diversity of AEs, but as common criteria used by scholars, physicians, and investigators all over the world, we also choose its definitions and systems to describe and classify these AEs. In clinical practice, recommendations should be flexible and individualized and must take into account many variables, such as disease phase, patients’ general conditions, and local medical facilities for handling severe emergencies.

**Table 2:** General algorithm for dose interruptions and reductions according to the severity of apatinib-associated adverse events.

Grade of AE According to CTCAE	Treatment	Modification of Dose
Grade 1 or 2	Unchanged	No modification
Grade 3	Interruption until recovery to < grade 2	First episode: try to restart at full dose Second episode: reduce the dose until the AE has reduced in severity by 1 grade
Grade 3 to 4	As there is a risk of death, consider a permanent reduction or interruption	

In some situations, grade 2 should lead to dose interruptions and reductions, e.g., wound complications and proteinuria. AE, adverse event; CTCAE, Common Terminology Criteria for Adverse Events.

### Hand-Foot Skin Reactions (HFSRs)

HFSRs (also known as palmar-plantar erythrodysesthesia) generally affect the palms and feet. When these become more severe, they are described as rash acneiform, which occurred in 37.8%–48.8% apatinib-treated patients. Typical manifestations are localized thick hyperkeratotic lesions that are surrounded by erythematous regions, which are always painful (22). Thus, although HFSRs are described differently in CTCAE, we usually incorporate these two definitions together. Occasionally, symptoms may also occur in other areas, such as the knees and elbows. HFSRs of any grade were the most common AEs associated with antiangiogenic TKIs in advanced sarcoma studies, occurring in 18% of patients in the pazopanib for metastatic soft-tissue sarcoma (PALETTE) trial (23) and 51% of patients treated with regorafenib for advanced osteosarcoma (5). It was also one of the most common AEs in sorafenib (24), lenvatinib (25) and cabozantinib (26) phase 3 advanced hepatocellular carcinoma studies (occurring in 21%, 27%, and 46% of the patients, respectively). Grade 3 HFSR, which can substantially impair patients’ ability to perform daily tasks and overall QoL, occurred in 3%–17% of patients in these studies (24–26). These lesions usually occur within the first 45−80 days of apatinib administration (6) and can negatively affect their QoL.

Proactive measures should be taken before skin lesion development, including oral administration of vitamin B tablets, frequent use of creams and moisturizers from the start of drug therapy, and avoidance of pressure and friction (i.e., plantar pads and wearing clothes with adequate room for the hands and feet) (27). Routine skin examination before treatment initiation and callus softening and removal are necessary before patients develop more severe conditions. Sun exposure and unprotected cold exposure should be avoided, and more aggressive strategies may include applying keratolytic creams that contain urea or salicylic acid (6%) on minor hyperkeratotic areas and locally spraying a solution of recombinant human basic endothelial or fibroblast growth factor or vitamin B on skin areas showing minor wear out (28).

In cases of HFSR developing into grade ≥2, consultation with a dermatologist is recommended. Nonsteroidal anti-inflammatory drugs (NSAIDs) (29) are suggested under some circumstances. The key strategies for HFSR management are to maintain or restore patient comfort to avoid reducing their QoL and influencing their daily activities and to maintain their treatment with apatinib for as long as possible. Dose reductions or treatment interruptions may be warranted in some scenarios, which usually leads to symptom alleviation within 2–7 days (6, 7, 13). Moisturizers containing 20%–40% urea, salicylic acid, ammonium lactate, or alpha hydroxyl acid may be used to soften and exfoliate hyperkeratotic and callus areas. Patients should consider using topical treatments, such as cortisone and 0.05% clobetasol, as well as antibiotic ointment to prevent infections (30). Sometimes local anesthetics are recommended for severe pain. In our experience, some Chinese herbal preparations for external use are clinically helpful, and these contain *Portulacaria*, *Geranium wilfordii* Maxim, *Rhizoma*, *Flos carthami*, and *Cortex phellodendri*, all of which have been formulated into medical products in some clinics in Beijing (6, 13).

### Stomatitis and Mucositis

Theoretically, stomatitis and mucositis are similar to HFSRs in that they are caused by capillary vasoconstriction due to antiangiogenic TKIs; they had an incidence of 18.9%–27.9% in apatinib-treated patients. However, to date, studies on treatments for VEGFR-2 inhibitor-induced mucosal toxicity are limited, and no randomized controlled trial data are currently available (31, 32). Furthermore, most published information specifically focuses on oral mucosal inflammation (stomatitis), ignoring inflammation of the nasal, anal, vaginal, or even whole alimentary tract mucosae. Information on the management of mucosal toxicity is usually available through document retrievals. Good oral hygiene is highly encouraged, including frequent teeth brushing, flossing and tongue cleaning with a soft brush (every 2–3 h for mild stomatitis; every 1–2 h for more severe symptoms) (33). Soft, nonirritating foods that are easy to chew and swallow are advised, and spicy, acidic, and salty foods should be avoided; however, this suggestion might exacerbate anorexia in individuals from some provinces in China, especially in Sichuan, Hubei and Hunan, where local people enjoy spicy and salty foods. Commercial mouthwashes containing alcohol should be avoided (27). Taking compound vitamin B tablets is also encouraged. Cleaning the rectal area with mild soap and water and using disposable wipes and a moisture-barrier ointment are recommended to prevent anal mucosal ulcers.

Because stomatitis/mucositis can become more severe than grade 2, it might be necessary to stop apatinib or reduce the dose. Topical anesthetics, mucosal coating agents and/or benzydamine HCl may be administered locally for pain alleviation (34–36). In cases of infections, topical or systemic antimicrobials may also be administered. Patients are suggested to go to specialist clinics for advice. For stomatitis/mucositis reaching grade 3, treatment with apatinib should be discontinued, and the patient is usually admitted to the hospital for supportive care (28). Cortisol paste should be suggested, and the dose of oral antibiotics should be increased. In addition, locally spraying a solution of recombinant human basic fibroblast growth factor that has been manufactured as an externally applied agent or vitamin B on the surfaces of ulceration may be considered. Some Chinese herbal preparations may also be used to promote ulcer wound healing (6). For anal ulcers, a Chinese patent medicine named Mayinglong hemorrhoid ointment, which contains traditional Chinese medicine anti-inflammatory ingredients and the pain reliever borneol, is extremely effective in improving patients’ QoL.

### Diarrhea

Although diarrhea is the most common AE experienced by patients receiving aa-TKIs, the underlying mechanism is not totally understood, although some experts believe that TKI-associated diarrhea is due to excessive chloride secretion (37). In the METEOR clinical trial, 63% of patients receiving cabozantinib experienced grade 1–2 diarrhea, and 11% experienced grade 3 diarrhea, of which the median onset of diarrhea was 4.9 weeks. This AE was the most common reason for cabozantinib dose reduction (16%) (38). Diarrhea was also the most common AE associated with sorafenib and apatinib, occurring in 26% and 19% of patients with advanced osteosarcoma, respectively (4, 6).

Generally, recommendations for TKI-associated diarrhea management are similar across tumor types. To decrease the risk of diarrhea, patients should be encouraged to maintain liquid volume, eat frequent small meals, and avoid lactose-containing food, high-fat products and alcohol (22). Using a stool diary to help identify foods that may trigger digestive problems may be useful for these patients. Patients are recommended to eat bananas, rice, potatoes, apple sauce, toast, and probiotics to help digestion. It is feasible and recommended for patients to refer to a dietician for evidence-based information to reduce the risk of diarrhea (27, 33). However, physicians must always be aware that dietary restrictions might have a negative impact on weight loss and hypokalemia in populations whose body weight maintenance can already be difficult.

In cases of diarrhea that cannot be managed by dietary control, loperamide (4 mg then 2 mg every 4 h, or even 2 mg every 2 h depending on personalized situations) can be prescribed (39). For patients experiencing diarrhea frequently, loperamide could also be orally taken preemptively 30 min before TKI treatment. It is suggested that patients drink enough liquids that contain water and electrolytes, and in appropriate situations, patients can even take atropine-diphenoxylate, codeine, or tincture of opium (40) for palliation, which have been suggested in the literature; however, in clinical practice, these are rarely used. Moreover, pancreatic enzyme supplements, such as azintamide and *Aspergillus oryzae* trypsin, may be considered to reduce diarrhea and improve digestion (29). It is usually suggested to take 1-2 pills three times after each meal every day as conventional medical care. At the same time, any concurrent gastrointestinal infections should be treated appropriately. If diarrhea continues to grade ≥ 3/4, then an absolute diet with parenteral nutrition and intravenous electrolytes and fluid supply is advised, and somatostatin analogs such as octreotide are commonly prescribed (21, 27, 33). Patients should be hospitalized or referred to a gastroenterologist, particularly when severe cramping, nausea and vomiting, fever, or even dehydration occurs (19).

### Anorexia and Weight Loss

Anorexia occurred in 35% of patients treated with apatinib and in 31% of patients treated with regorafenib in trials for advanced osteosarcoma (5, 6). Weight loss was experienced by 11%–35% of these patients. Severe hypokalemia usually follows anorexia and weight loss. Although both AEs are common and closely related, patients can also experience weight loss as a result of diarrhea, with or without anorexia, which is more subjective according to the patient’s complaints.

In our experience, appetite and weight should be monitored in each treatment cycle. Consuming nutritionally endowed foods with high calorific value as well as high-protein foods and snacking throughout the day (on foods such as eggs, poultry, fish, honey, cheese, and gelatin) is encouraged; foods that could cause gastrointestinal events should be limited or avoided (22, 30, 40). Consulting an endocrinologist in some circumstances is necessary because of the presence of underlying conditions, including hypothyroidism and low testosterone in men (22, 30). However, similar to that in patients presenting with fatigue, the administration of corticoids to stimulate appetite is also controversial. For patients who experience anorexia, stimulants such as megestrol acetate might be suggested, but they are rarely administered in China because of the restrictions on physicians’ prescriptions (41).

### Blood Bilirubin Increases

Few reports have described increases in blood bilirubin with aa-TKI treatment (42). However, in two trials, nearly 20% of the patients showed an increase in bilirubin levels after the long-term use of apatinib, and chronic cholestatic cholecystitis might develop, causing severe abdominal pain and drug interruption. Thus, for those with increased bilirubin, various initial work-ups to exclude infections or other etiologies should be performed. Ursodeoxycholic acid could be considered in individuals with cholestatic drug-induced liver injury (DILI) (43). When DILI occurs, it is recommended to detect variables reflecting hepatic uptake, conjugation, or excretion as well as biliary obstruction and/or hemolysis. Isolated elevation of the conjugated fraction does not truly signify DILI. When these factors are associated with an increase in alanine aminotransferase (ALT) (Hy’s law) (44), we assume DILI might occur. Corticosteroids are frequently administered to define DILI (such as that associated with liver dysfunction) (44). When acute liver failure occurs, a hepatologist should be consulted for the need for artificial liver and liver transplantation.

### Wound Complications

Approximately 11% of apatinib-treated osteosarcoma patients may develop wound problems (6). Currently, there are no evidence-based guidelines on perioperative management and drug suspension time in patients treated with TKIs due to the lack of formal clinical data. Impaired wound healing is a potential problem following treatment with TKIs, such as axitinib, cabozantinib, pazopanib, ponatinib, regorafenib, sorafenib, sunitinib, and vandetanib (45). In terms of frequency, there are no data available because no formal studies have been conducted to assess the potential impact of any TKI on wound healing. There is an urgent demand for unified retrospective studies to analyze existing data from various clinical trials investigating wound complications following all kinds of operations in patients receiving TKIs. We summarized all the half-lives of aa-TKIs and the recommendations for drug interruption intervals for selective surgeries (**Table 3**) according to the drug labels, which in our opinion was not reliable in real-life scenarios due to tumor progression after long drug interruption time intervals. It is suggested from the package insert that apatinib should be stopped at least 30 days prior to scheduled surgery, which is usually not executable due to “tumor rebound” after therapy discontinuation for such a long period of time. However, the appropriate time for stopping apatinib treatment before surgery should be established with caution based on the location and complexity of elective surgery, and there are diverse opinions from different medical institutions in China. Collaborations between surgeons and oncologists on debridement and dressing changes in patients are encouraged.

**Table 3:** Summary of regulatory recommendations concerning the use of TKIs prior to surgery.

TKI	Half-Life (h)	Monitoring Recommendations^a^
Apatinib	7.8	Treatment should be stopped at least 30 days prior to scheduled surgery
Anlotinib	116	Treatment should be stopped at least 4 weeks prior to scheduled surgery
Pazopanib	31	Treatment should be stopped at least 7 days prior to scheduled surgery
Regorafenib	28 (parent); 25–51 (metabolite)	Treatment should be stopped at least 2 weeks prior to scheduled surgery
Sorafenib^b^	25–48	Interruption is recommended in patients undergoing major surgical procedures
Sunitinib^b^	40–60 (parent); 80–110 (metabolite)	Temporary interruption is recommended for precautionary reasons in patients undergoing major surgical procedures
Cabozantinib	55	Treatment should be stopped at least 28 days prior to scheduled surgery
Axitinib	2.5–6.1	Treatment should be stopped at least 24 h prior to scheduled surgery

a According to the latest labels for detailed recommendations on dose modifications before elective surgery.

b Use with caution in patients at risk of gastrointestinal perforation or fistula, especially after gastrointestinal surgeries. TKI, tyrosine kinase inhibitor.

### Hypertension

Hypertension is a frequent AE associated with VEGF pathway inhibition, with any-grade hypertension occurring in 9%–30% of the population and grade ≥3 hypertension occurring in 0%–24% of advanced osteosarcoma patients (4–6). In apatinib-treated children and young adults, the incidence of hypertension was 18.9%–23.3% (4–6). Some studies have also suggested that hypertension may be utilized as a marker for favorable clinical outcomes (46). However, in most adolescents and young adults, hypertension is not as severe as that observed in adult patients treated with other aa-TKIs (6).

Blood pressure should be monitored and under control before initiating TKI treatment and should be regularly under surveillance for the first few months of treatment; blood pressure monitoring could be conducted during clinic visits. However, in our opinion, if appropriate, it is preferable for patients to self-monitor their blood pressure at home multiple times a day (47). If blood pressure is stable, then the monitoring frequency may be subsequently reduced. To manage hypertension, current guidelines recommend initial antihypertensive treatment with angiotensin-converting enzyme inhibitors (ACEIs), angiotensin receptor blockers (ARBs), and beta blockers (22, 27–29, 41, 46). Sometimes, ACEIs might cause severe cough in adults, and thus, these agents may be substituted with ARBs, as these agents could also reduce proteinuria. In addition, some studies have shown that the combination of apatinib and ACEIs may have a synergistic effect in controlling tumor growth (48). Calcium channel blockers (CCBs) may also be administered (33, 49); however, caution should be taken in selecting the specific agent to use. Diltiazem and verapamil are nondihydropyridine CCBs that inhibit CYP3A4, and similar agents should be avoided with apatinib due to potential drug–drug interactions (50). For difficult-to-control hypertension, we recommend three standard antihypertensive agents, although ACEIs and ARBs should not be combined. Caution should be taken when using thiazide diuretics owing to the risk of diarrhea, electrolyte loss, and QT prolongation (51). The BP goal for patients older than 60 years could be 150/90 mmHg, and for younger patients, it should be 140/90 mmHg. When antihypertensives are not effective, TKI dose reductions or interruptions may be necessary (28, 29, 51). In persistent cases, TKI discontinuation should be considered (3, 6, 15).

### Proteinuria

Proteinuria is usually difficult to notice, especially in patients who do not have any obvious complaints, and it is also hard to address. In the two trials, proteinuria usually occurred 158 (95% CI: 12–186) days after the initiation of apatinib treatment in 11% of the cohort (6, 7). It is recommended to regularly monitor proteinuria monthly, if possible. In China, some Chinese patent medicines containing musk mallow and *Paecilomyces hepiali Chen* may be considered to prevent patients with proteinuria of less than grade 2+ from developing a more severe grade (6, 12, 13). Twenty-four-hour urine protein quantitation is advised when patients’ routine urine analysis indicates proteinuria. ACEIs or ARBs may also be considered to reduce proteinuria (22, 30). Long-term proteinuria might cause renal failure and uncontrollable hypoproteinemia (52).

### Pneumothorax

The development of cavities for pulmonary nodules is evidence of a treatment response to TKIs and is also a risk factor for pneumothorax (53). As pneumothorax is a potentially fatal complication due to apatinib-based treatment, its risk and treatment interruption hazard should be balanced with discretion. In prospective trials of aa-TKIs for advanced osteosarcoma, pneumothorax developed in 3%, 0%, and 32% of patients who received sorafenib (4), regorafenib (5), and apatinib, respectively (6). In a retrospective report evaluating the combination of sorafenib, bevacizumab, and low-dose cyclophosphamide in children’s solid tumors, an unexpectedly high incidence of pneumothorax was observed, with an incidence of 25% (11/44) (54). It is hypothesized that the use of VEGF blockers induces pulmonary nodule necrosis secondary to vascular constrictions, which causes subsequent necrosis and results in pathologic pneumothorax (55).

There is no prophylactic treatment for pneumothorax. The risk factors for spontaneous pneumothorax are related to pulmonary metastasis, the majority of which are located at the pleural or peripheral zone of the lung. Although some patients who have pneumothorax during treatment are asymptomatic, most of them have chief complaints of chest pain and dyspnea (54). Appropriate measures for pneumothorax include observation without intervention, observation with oxygen inhalation, needle aspiration, insertion of thoracostomy tube to exhaust gas, or even invasive thoracoscopy or thoracotomy (56). Although treatment options usually depend on patients’ clinical symptoms and the severity of the pneumothorax, the management of pneumothorax, especially in those with osteosarcoma, is challenging, and to date, there have been no established guidelines for dealing with pneumothorax induced by TKI usage. Furthermore, establishing a consolidated and publicly accepted approach is difficult, as the data in osteosarcoma are from small-sample studies. This AE usually requires interruption of apatinib but causes disease progression. Thus, we recommend the use of a pigtail catheter or chest tube to evacuate pneumothorax and then the use of chemical or mechanical pleurodesis. Pleurodesis is an efficient method for the treatment of recurrent pneumothorax, as extensive studies on various agents, such as talc, silver nitrate, bleomycin, tetracycline, mitoxantrone, mepacrine, *Corynebacterium parvum*, and povidone, have shown that these adhere to the visceral and parietal pleura (57–59). From our experience, it is recommended to use *Staphylococcus aureus* byproducts for pleurodesis. Usually, after three to five injections of a combination solution of *S. aureus* byproducts and local anesthetics, pleurodesis is performed without interruption of apatinib; however, during this procedure, patients usually experience fever and chest pain, which should be managed with symptomatic treatment.

### Nausea and Vomiting

In the phase 3 clinical trials discussed here, the nausea rates were 24% for sorafenib, 17% for regorafenib, 20% for lenvatinib, 23.3% for apatinib and 31% for cabozantinib (24–26, 60). Vomiting occurred in 15%, 13%, 16%, and 26% of the patients in these trials. In each study, ≤2% of patients experienced grade ≥ 3 nausea or vomiting (26).

It has been suggested that avoiding chocolate, caffeine, alcohol, and nicotine may be helpful (**Table 4**) (29). Antiemetics, such as metoclopramide and levosulpiride, may alleviate symptoms. 5-HT3 antagonists have been recommended for only NK1 receptor antagonists (e.g., dexamethasone) to avoid CYP3A4 modulation. Ondansetron and granisetron should be used with caution owing to potential interactions with apatinib, causing QTc prolongation (50, 61). In addition, we recommend a healthy lifestyle, dietary modifications and prophylactic use of proton-pump inhibitors to avoid nausea and vomiting.

**Table 4:** Management of common adverse events associated with apatinib for advanced osteosarcoma.

Adverse Event	Recommended Management Strategies
HFSR	Prophylactic management (for grade 1 and 2): Skin examination before initiation of treatment, softening and removal of calluses; Protection against pressure and friction (i.e., plantar pads and wearing clothes with adequate room for the hands and feet); Frequent local administration of creams and moisturizers before treatment; Orally administration of compound vitamin B tablets; Sun exposure and unprotected cold exposure should be avoided; Keratolytic creams can be used on hyperkeratotic areas; Locally spray a solution of recombinant human basic endothelial growth factor or vitamin B on skin with minor wear out.
	AE management: Preventative measures should be continued; Moisturizers containing 20–40% urea, salicylic acid, ammonium lactate or alpha hydroxyl acid may be used to soften and exfoliate hyperkeratotic and callused areas (for grade 2); Consider using topical treatments such as cortisone and 0.05% clobetasol (for grade 3); Consider using antibiotic ointment to prevent infection (for grade 3); If HFSR is grade ≥2, a dermatologist consultation is suggested and local anesthetics are recommended for severe pain; Consider Chinese herbal preparation solution for external use (for all grades).
Stomatitis and Mucositis	Prophylactic management (for grade 1): Appropriate oral hygiene before treatment initiation; Avoid spicy, acidic, hard or hot foods and drinks; Oral administration of compound vitamin B tablets; Clean and dry the rectal area with mild soap and water and use a moisture-barrier ointment locally after cleaning.
	AE management: A high-energy diet and adequate fluid (for grade 2 and 3); In cases of severe mucositis, enteral or parenteral nutrition is recommended (for grade 3); Use gentle mouthwashes after meals and topical anesthetics or use products containing hyaluronic acid in composition locally (for grade 2 and 3); Consider using Chinese herbal preparation solutions that contain Portulacaria, geranium wilfordii maxim, rhizoma, Flos carthami, and cortex phellodendri locally (for grade 2 and 3); Locally spray a solution of recombinant human basic endothelial growth factor or vitamin B on stomatitis (for grade 2 and 3).
Diarrhea	Prophylactic management (for grade 1 and 2): Anti-diarrhea diet (avoid fiber, fat and acrimony excitant food); Use a stool diary to help identify foods that may trigger digestive problems; Consumption of bananas, rice, potatoes, apple sauce, toast and probiotics may be helpful; Caffeine, alcohol, spicy or fatty foods, dairy products and foods high in insoluble fiber should be avoided.
	AE management: In cases that cannot be managed by dietary changes, loperamide (4 mg then 2 mg every 4 h) may be prescribed. For patients who frequently experience diarrhea, loperamide may also be taken pre-emptively, 30 min before TKI treatment (for grade 3); Intensive oral rehydration containing water and electrolytes (for grade 2 and 3); Consider treatment with atropine-diphenoxylate, if appropriate (for grade 3); A pancreatic enzyme supplement might be considered to reduced diarrhea and improve digestion (for grade 2 and 3); Any concurrent gastrointestinal infection should be treated appropriately (for grade ≥ 3); If diarrhea is grade ≥ 3/4, absolute diet with parenteral nutrition and intravenous electrolytes and fluid supply is advised, and somatostatin analogs such as octreotide are commonly prescribed (for grade ≥ 3); Hospitalization or referral to a gastroenterologist should also be advisable, particularly in the event of severe cramping, nausea and vomiting, fever, or dehydration (for grade ≥ 3); Concomitant lactulose dose reduction may be necessary (for grade ≥ 3).
Fatigue	Prophylactic management (for grade 1 and 2): Other potentially treatable coexisting causes of fatigue, such as anemia, diarrhea, nausea, hypothyroidism, hypokalemia, and insomnia, should be corrected in case of deteriorating fatigue; For patients who are fit enough, daily exercise such as walking or weight-bearing exercises may be useful.
	AE management: Psychostimulants, such as caffeine, or methylphenidate or modafinil for more severe cases, may be considered; however, care should be taken when prescribing modafinil owing to potential interactions with apatinib (for grade 3); Based on preventative measures, steroid cortisol could be considered as a hormone supplement (for grade 3); Taking apatinib in the evening rather than the morning may reduce daytime fatigue (for all grades).
Blood Bilirubin Increases	Prophylactic management (for all grades): Various initiate work-up for competing etiologies; Ursodeoxycholic acid could be considered in individuals with cholestatic DILI.
	AE management: Consult with hepatologists for more advice and stopping apatinib (for grade ≥ 3); Corticosteroids are frequently administered to patients with certain DILI (for grade ≥ 3); Cholestyramine can be administered to patients with acute liver injury (for grade ≥ 2); When acute liver failure happens, considering artificial liver and liver transplantation (for grade ≥ 4).
TSH Increases	Prophylactic management (for grade 1): Levothyroxine supplement after consultation with an endocrinologist.
	AE management: Hospitalization with supply of hormones, electrolytes and fluids (for all grades); Monitoring patients’ vital signs (for all grades).
Anorexia and Weight loss	Prophylactic management (for all grades): Encourage patients to consume nutritious, high-calorie foods and to eat snacks throughout the day; Limit/avoid foods that could cause gastrointestinal events; Appetite and weight should be monitored in each treatment cycle.
	AE management: Appetite stimulants such as dronabinol or megestrol acetate should be considered (for grade ≥ 3); Any underlying nausea should be treated (for grade 2 and 3); High-calorie diet and dietary supplements should be recommended and nasogastric feedings should be considered (for grade ≥ 3); The presence of underlying conditions, including hypothyroidism, low testosterone in men and so on, should be managed well (for all grades); Be aware of asthenia-anorexia-cachexia syndrome, characterized by weight loss, weakness and fatigue. This condition can be treated with corticosteroids, although these may only be effective in the short term (for grade ≥ 3).
Hyperlipidemia	Prophylactic management (for all grades): Test the lipid profile at baseline and during the course of treatment.
	AE management: In the event of persistent hypercholesterolemia (higher than 6.2 mmol/L, considered high risk according to AACE guidelines), add an appropriate statin, which should be decided with caution because of cytochrome P450 (CYP3A) (for grade 2).
Wound Complications	Prophylactic management: Selecting an appropriate interval for stopping the use of apatinib before surgery is advised according to the location and complexity of the elective surgery (grade 1 and 2); Postoperatively, in the presence of advanced cancer, drug-induced impaired wound healing and organ perforation should be taken into account. The timing of reinitiation of therapy following a major surgical intervention should be based upon clinical judgment of recovery from surgery (grade 1 and 2).
	AE management: The surgeon and oncologist should collaborate on debridement and dressing changes for the patient (grade 3); Therapy should be discontinued in patients with wound dehiscence (for grade ≥3).
Hypertension	Prophylactic management (for all grades): Blood pressure should be controlled before initiating TKI treatment; Blood pressure should be monitored regularly for the first few months of treatment.
	AE management: ACEIs, ARBs or beta blockers should initially be encouraged to be used to treat hypertension (grade 1 and 2); Provide up to three standard antihypertensive agents, but do not combine ACEIs and ARBs (grade 3); Calcium channel blockers may be considered, but careful selection is necessary to avoid interactions with TKIs (avoiding CYP3A4 inhibitor/inducers, e.g., verapamil and diltiazem) (grade 3); Caution should be taken when using thiazide diuretics owing to the risk of diarrhea (grade 3).
Proteinuria	Prophylactic management: Monitor proteinuria regularly, monthly if possible (for all grades); Consider some Chinese patent medicines containing musk mallow and Paecilomyces hepiali (grade 1 and 2).
	AE management: Dose reductions and monitoring at the clinic multiple times per week (for grade ≥2); Considering ACEIs or ARBs to reduce proteinuria (grade 2 and 3).
Pneumothorax	Prophylactic management: Closely monitoring chest plain or dyspnea (for all grades).
	AE management: Consider using a pigtail catheter or chest tube to evacuate the pneumothorax and later using chemical or mechanical pleurodesis (grade 2, 3 and 4); In some severe situations, consider using video-assisted thoracoscopic surgery and chemical pleurodesis for patients with first time pneumothorax (grade 3 and 4); For pleurodesis, highly agglutinative staphylococcin is preferred (grade 2 and 3).
Hypokalemia	Prophylactic management (for all grades): Monitor serum potassium after every treatment cycle, especially in those with anorexia; An oral potassium replacement should be provided to patients with signs of hypokalemia.
	AE management: An oral or intravenous potassium replacement should be considered for those with mild or moderate hypokalemia (for grade ≥2); Hospitalization with an intravenous potassium supply and close monitoring with serum potassium and electrocardiogram (for grade ≥3).
Nausea and Vomiting	Prophylactic management: Chocolate, caffeine, alcohol and nicotine should be avoided (for all grades); Antiemetics may be provided prophylactically (grade 2 and 3).
	AE management: Pharmacological treatment with metoclopramide or levosulpiride may be considered (grade 2 and 3); 5-HT_3_ antagonists are recommended over the use of NK1 receptor antagonists, dexamethasone, or nabilone to avoid CYP3A4 modulation; ondansetron and granisetron should be used with caution owing to potential interactions with apatinib (grade 2 and 3); Consider guidelines for GERD, including lifestyle and dietary modifications and the use of proton-pump inhibitors (for grade ≥2).

AACE, American Association of Clinical Endocrinologists; ACEIs, angiotensin-converting enzyme inhibitors; AE, adverse event; ALT, alanine aminotransferase; ARBs, angiotensin receptor blockers; NCI-CTCAE, National Cancer Institute Common Terminology Criteria for Adverse Events; DILI, drug-induced liver injury; GERD, gastroesophageal reflux disease; HFSR, hand–foot skin reaction; TKI, tyrosine kinase inhibitor; TSH, thyroid-stimulating hormone.

### Myelosuppression

Myelosuppression developing during apatinib treatment of osteosarcoma is rare. The hematological toxicity of TKIs is usually mild, persistent, dose-dependent, and reversible upon cessation or dose reduction; TKIs casually affect leukocytes, erythrocytes and thrombocytes to varying degrees (62). We compared the hematopoietic toxicities of different TKIs with dose adjustments for neutropenia and thrombocytopenia (**Table 5**).

**Table 5:** Management of hematopoietic toxicity.

TKI	Starting Dose	Hematopoietic Toxicity	Dose Adjustments for Neutropenia and Thrombocytopenia
Sorafenib	400 mg twice daily	ANC <1.0 × 10^9^/L and/or platelets <50 × 10^9^/L	400 mg once daily
Dasatinib	100 mg once daily	ANC <0.5 × 10^9^/L and/or platelets <50 × 10^9^/L	Stop dasatinib until ANC >1.0 × 10^9^/L and platelets >50 × 10^9^/L, resume the original starting dose
Imatinib	400 mg twice daily	ANC <1.0 × 10^9^/L and/or platelets <50 × 10^9^/L	200 mg twice daily
Pazopanib	800 mg once daily	ANC <1.0 × 10^9^/L and/or platelets <50 × 10^9^/L	600 mg once daily
Regorafenib	160 mg once daily 3 w^a^ on, 1 w off	ANC <1.0 × 10^9^/L and/or platelets <50 × 10^9^/L	120 mg once daily 3 w on, 1 w off
Apatinib	500 mg once daily	ANC <1.0 × 10^9^/L and/or platelets <50 × 10^9^/L	250 mg once daily
Anlotinib	12 mg once daily 2 w on, 1 w off	ANC <1.0 × 10^9^/L and/or platelets <50 × 10^9^/L	10 mg once daily 2 w on, 1 w off

a w, week; TKIs, tyrosine kinase inhibitors.

## Conclusions

Patients who receive apatinib-based therapy should be educated on the commonly seen AE manifestations and prophylactic and symptomatic measures and should routinely arrange follow-ups to obtain timely and appropriate supportive care and be monitored for rare, asymptomatic events. Otherwise, these AEs may immensely influence patients’ QoL and increase the risk of dose reduction or drug discontinuation, which in turn may compromise clinical outcomes. In addition, the interventions being described should be better assessed in prospective trials to evaluate whether these interventions may improve symptoms and to evaluate the dose delivered to patients.

## Data Availability Statement

Publicly available datasets were analyzed in this study. This data can be found here: https://www.ncbi.nlm.nih.gov/pmc/articles/PMC7223462/. The datasets are available from the corresponding author upon reasonable request. We promised to cover patients’ data confidentially. But patients’ data will be made disguisedly available, including data dictionaries, for approved data sharing requests. Individual data will be shared that underlie the results reported in this Article, after de-identification and normalization of information (text, tables, figures, and appendices). Proposals should be directed to xie.lu@hotmail.com. To gain access, data requestors will need to sign a data access agreement.

## Author Contributions

WG, LX, and JXu made substantial contributions to the conception and design of the study. LX and JXu drafted the article. All authors contributed to the article and approved the submitted version.

## Funding

Financial support was provided by the National Natural Science Foundation of China (grant no. 81572633).

## Conflict of Interest

The authors declare that the research was conducted in the absence of any commercial or financial relationships that could be construed as a potential conflict of interest.

## Supplementary Material

Supplementary Table 1 PRISMA flow diagram.
