# Supplementary material for: Management of Apatinib-Related Adverse Events in Patients With Advanced Osteosarcoma From Four Prospective Trials: Chinese Sarcoma Study Group Experience
Source: Front Oncol. 2021 Jul 22;11:696865. doi: 10.3389/fonc.2021.696865 (PMC8339966; doi:10.3389/fonc.2021.696865)
Supplement: Supplementary Table 1 — PRISMA flow diagram. [file Table_1.docx]

Supplementary table 1: Detailed clinical manifestation different grades of CTCAE 5.0^1^

| Adverse events | Grade 1 | Grade 2 | Grade 3 | Grade 4 | Grade 5 | Definition |
| --- | --- | --- | --- | --- | --- | --- |
| Hand foot Skin Reactions (HFSRs) | Minimal skin changes or dermatitis (e.g., erythema, edema, or hyperkeratosis) without pain | Skin changes (e.g., peeling, blisters, bleeding, fissures, edema, or hyperkeratosis) with pain; limiting instrumental ADL^2^ | Severe skin changes (e.g., peeling, blisters, bleeding, fissures, edema, or hyperkeratosis) with pain; limiting self care ADL | ND^3^ | ND | A disorder characterized by redness, marked discomfort, swelling, and tingling in the palms of the hands or the soles of the feet. Also known as Hand-Foot Syndrome or palmar-plantar erythrodysesthesia syndrome. |
| Stomatitis and Mucositis | Asymptomatic or mild symptoms; intervention not indicated | Moderate pain or ulcer that does not interfere with oral intake; modified diet indicated | Severe pain; interfering with oral intake | Life-threatening consequences; urgent intervention indicated | Death | A disorder characterized by ulceration or inflammation of the alimentary tract mucosal. |
| Diarrhea | Increase of <4 stools per day over baseline; mild increase in ostomy output compared to baseline | Increase of 4 - 6 stools per day over baseline; moderate increase in ostomy output compared to baseline; limiting instrumental ADL | Increase of >=7 stools per day over baseline; hospitalization indicated; severe increase in ostomy output compared to baseline; limiting self care ADL | Life-threatening consequences; urgent intervention indicated | Death | A disorder characterized by an increase in frequency and/or loose or watery bowel movements. |
| Anorexia | Loss of appetite without alteration in eating habits | Oral intake altered without significant weight loss or malnutrition; oral nutritional supplements indicated | Associated with significant weight loss or malnutrition (e.g., inadequate oral caloric and/or fluid intake); tube feeding or TPN indicated | Life-threatening consequences; urgent intervention indicated | Death | A disorder characterized by a loss of appetite. |
| Weight Loss | 5 to <10% from baseline; intervention not indicated | 10 - <20% from baseline; nutritional support indicated | >=20% from baseline; tube feeding or TPN indicated | ND | ND | A finding characterized by a decrease in overall body weight; for pediatrics, less than the baseline growth curve. |
| Blood Bilirubin Increased | >ULN^4^ - 1.5 x ULN if baseline was normal; > 1.0 - 1.5 x baseline if baseline was abnormal | >1.5 - 3.0 x ULN if baseline was normal; >1.5 - 3.0 x baseline if baseline was abnormal | >3.0 - 10.0 x ULN if baseline was normal; >3.0 - 10.0 x baseline if baseline was abnormal | >10.0 x ULN if baseline was normal; >10.0 x baseline if baseline was abnormal | ND | A finding based on laboratory test results that indicate an abnormally high level of bilirubin in the blood. Excess bilirubin is associated with jaundice. |
| Wound Complication | Observation only; topical intervention indicated | Bedside local care indicated | Operative intervention indicated | Life-threatening consequences | Death | A finding of development of a new problem at the site of an existing wound. |
| Hypertension | Adult: Systolic BP 120 - 139 mm Hg or diastolic BP 80 - 89 mm Hg; Pediatric: Systolic/diastolic BP >90th percentile but< 95th percentile; Adolescent: BP ≥120/80 even if < 95th percentile | Adult: Systolic BP 140 - 159 mm Hg or diastolic BP 90 - 99 mm Hg if previously WNL; change in baseline medical intervention indicated; recurrent or persistent (>=24 hrs); symptomatic increase by >20 mm Hg (diastolic) or to >140/90 mm Hg; monotherapy indicated initiated; Pediatric and adolescent: Recurrent or persistent (>=24 hrs) BP >ULN; monotherapy indicated; systolic and /or diastolic BP between the 95th percentile and 5 mmHg above the 99th percentile; Adolescent: Systolic between 130-139 or diastolic between 80-89 even if < 95th percentile | Adult: Systolic BP >=160 mm Hg or diastolic BP >=100 mm Hg; medical intervention indicated; more than one drug or more intensive therapy than previously used indicated; Pediatric and adolescent: Systolic and/or diastolic > 5 mmHg above the 99th percentile | Adult and Pediatric: Life-threatening consequences (e.g., malignant hypertension, transient or permanent neurologic deficit, hypertensive crisis); urgent intervention indicated | Death | A disorder characterized by a pathological increase in blood pressure. |
| Proteinuria | 1+ proteinuria; urinary protein ≥ULN - <1.0 g/24 hrs^5^ | Adult: 2+ and 3+ proteinuria; urinary protein 1.0 - <3.5 g/24 hrs; Pediatric: Urine P/C (Protein/Creatinine) ratio 0.5 - 1.9 | Adult: Urinary protein >=3.5 g/24 hrs; 4+ proteinuria; Pediatric: Urine P/C (Protein/Creatinine) ratio >1.9 | ND | ND | A disorder characterized by laboratory test results that indicate the presence of excessive protein in the urine. It is predominantly albumin, but also globulin. |
| Pneumothorax | Asymptomatic; clinical or diagnostic observations only; intervention not indicated | Symptomatic; intervention indicated | Sclerosis and/or operative intervention indicated; hospitalization indicated | Life-threatening consequences; urgent intervention indicated | Death | A disorder characterized by abnormal presence of air in the pleural cavity resulting in the collapse of the lung. |
| Nausea | Loss of appetite without alteration in eating habits | Oral intake decreased without significant weight loss, dehydration or malnutrition | Inadequate oral caloric or fluid intake; tube feeding, TPN, or hospitalization indicated | ND | ND | A disorder characterized by a queasy sensation and/or the urge to vomit. |
| Vomiting | Intervention not indicated | Outpatient IV hydration; medical intervention indicated | Tube feeding, TPN, or hospitalization indicated | Life-threatening consequences | Death | A disorder characterized by the reflexive act of ejecting the contents of the stomach through the mouth. |
| Myelosuppression | <LLN^6^ - 3000/mm3; <LLN - 3.0 x 10e9 /L | <3000 - 2000/mm3; <3.0 - 2.0 x 10e9 /L | <2000 - 1000/mm3; <2.0 - 1.0 x 10e9 /L | <1000/mm3; <1.0 x 10e9 /L | ND | A finding based on laboratory test results that indicate a decrease in number of white blood cells in a blood specimen. |
| Fatigue | Fatigue relieved by rest | Fatigue not relieved by rest; limiting instrumental ADL | Fatigue not relieved by rest, limiting self care ADL | ND | ND | A disorder characterized by a state of generalized weakness with a pronounced inability to summon sufficient energy to accomplish daily activities. |
| TSH increased | TSH increased and no intervention initiated | ND | ND | ND | ND | A disorder characterized by an increase in thyroid stimulating hormone. |
| Hyperlipidemia | Requiring diet changes | Requiring pharmaceutical intervention | Hospitalization; pancreatitis | Life-threatening consequences | ND | A disorder characterized by laboratory test results that indicate an elevation in the concentration of lipids in blood. |
| Hypokalemia | <LLN - 3.0 mmol/L | Symptomatic with <LLN - 3.0 mmol/L; intervention indicated | <3.0 - 2.5 mmol/L; hospitalization indicated | <2.5 mmol/L; life-threatening consequences | ND | A disorder characterized by laboratory test results that indicate a low concentration of potassium in the blood. |

^1^An Adverse Event (AE) is any unfavorable and unintended sign (including an abnormal laboratory finding), symptom, or disease temporally associated with the use of a medical treatment or procedure that may or may not be considered related to the medical treatment or procedure. An AE is a term that is a unique representation of a specific event used for medical documentation and scientific analyses.

All these descriptions are according to Common Terminology Criteria for Adverse Events (CTCAE) Version 5.0, Published: November 27, 2017, U.S. DEPARTMENT OF HEALTH AND HUMAN SERVICES;

^2^Activities of Daily Living (ADL);

^3^ND: not defined;

^4^ULN: upper limit of Nomal;

^5^hrs: hours;

^6^LLN: lower limits of normal.
